# Bone Morphogenetic Protein 7 (BMP-7) Influences Tendon-Bone Integration *In Vitro*

**DOI:** 10.1371/journal.pone.0116833

**Published:** 2015-02-02

**Authors:** Tim Schwarting, Philipp Lechler, Johannes Struewer, Marius Ambrock, Thomas Manfred Frangen, Steffen Ruchholtz, Ewgeni Ziring, Michael Frink

**Affiliations:** 1 Department of Trauma, Hand- and Reconstructive Surgery, University Hospital Giessen and Marburg, Marburg, Germany; 2 Department of Orthopaedics and Rheumatology, University Hospital Giessen and Marburg, Marburg, Germany; Université de Lyon — Université Jean Monnet, FRANCE

## Abstract

**Introduction:**

Successful graft ingrowth following reconstruction of the anterior cruciate ligament is governed by complex biological processes at the tendon-bone interface. The aim of this study was to investigate in an *in vitro* study the effects of bone morphogenetic protein 7 (BMP-7) on tendon-bone integration.

**Materials and Methods:**

To study the biological effects of BMP-7 on the process of tendon-bone-integration, two independent *in vitro* models were used. The first model involved the mono- and coculture of bovine tendon specimens and primary bovine osteoblasts with and without BMP-7 exposure. The second model comprised the mono- and coculture of primary bovine osteoblasts and fibroblasts. Alkaline phosphatase (ALP), lactate dehydrogenase (LDH), lactate and osteocalcin (OCN) were analyzed by ELISA. Histological analysis and electron microscopy of the tendon specimens were performed.

**Results:**

In both models, positive effects of BMP-7 on ALP enzyme activity were observed (p<0.001). Additionally, similar results were noted for LDH activity and lactate concentration. BMP-7 stimulation led to a significant increase in OCN expression. Whereas the effects of BMP-7 on tendon monoculture peaked during an early phase of the experiment (p<0.001), the cocultures showed a maximal increase during the later stages (p<0.001). The histological analysis showed a stimulating effect of BMP-7 on extracellular matrix formation. Organized ossification zones and calcium carbonate-like structures were only observed in the BMP-stimulated cell cultures.

**Discussion:**

This study showed the positive effects of BMP-7 on the biological process of tendon-bone integration *in vitro*. Histological signs of improved mineralization were paralleled by increased rates of osteoblast-specific protein levels in primary bovine osteoblasts and fibroblasts.

**Conclusion:**

Our findings indicated a role for BMP-7 as an adjuvant therapeutic agent in the treatment of ligamentous injuries, and they emphasized the importance of the transdifferentiation process of tendinous fibroblasts at the tendon-bone interface.

## Introduction

Successful reconstruction of the anterior cruciate ligament (ACL) is influenced by various mechanical and biological factors. Whereas the majority of biomechanical and clinical studies have focused on optimization of the operative insertion of the transplant, the role of the biological processes at the tendon-bone interface remains to be elucidated [1]. The interface region between the bone and the graft is characterized by the existence of four histological zones, each with a distinct composition of cell types and extracellular matrix (ECM) structures (tendon, non-mineralized fibrocartilage, mineralized fibrocartilage and subchondral bone). The process of tendon-bone integration is governed by the dynamic spatial distribution of these subtypes of connective tissue resulting from the interactions between tenocytes and osteoblasts, causing the subsequent transdifferentiation processes of cells and ECM [2].

Bone morphogenetic proteins (BMPs) are important members of the super-family of tissue growth factors, representing a group of mediators that regulate cellular growth and differentiation [3]. The biological effects of BMPs are mediated by the regulation of various target genes, as analyzed by gene expression profiling [4]. More than 20 subtypes of BMPs have been identified [5], whereas a limited number of them have conveyed osteoinductive effects by differentiating mesenchymal stem cells into chondro- and osteoblasts [6]. In addition to its key role in osteogenesis, BMP-7 is a crucial factor in bone homeostasis and vertebral development [7]. Recombinant BMP-7 is an established adjuvant therapeutic measure for the treatment of non-unions following fractures and osteotomies [8].

A number of studies have revealed an important role of BMPs in bone-tendon integration by promoting healing and improving mechanical strength [9]; however, the exact biological mechanisms of BMPs in the physiological processes of bone tendon ingrowth have not been completely clarified. This study aimed to analyze the effects of BMP-7 on the interaction between osteoblasts and tendon grafts *in vitro*.

## Materials and Methods

Unless otherwise stated, the chemicals were obtained from Sigma-Aldrich (Taufkirchen, Germany). Animals were slaughtered for the meat-processing industry, specimens were collected directly postmortem from a nationally licensed abattoir (Meier III, Marburg, Germany). According to §7 of the German Protection of Animals Act (TierSchG) no animal experiments were performed, thus not requiring the approval of local authorities. All experiments were performed in triplicates. Mean values and standard deviation represent the data from three independent experiments.

### Cell and culture conditions

The cells were cultivated in a CO_2_ incubator (HeraCell 150i, Thermo Fishers Scientific, Waltham, MA USA) at 37°C in 5% CO_2_-humidified air. The tissue preparation and subculturing were performed under laminar flow in a sterile cabinet (Bio-II-A, Telstar, Terrassa, Spain). The living cells were counted using a CASY 1 cell counter, according to the manufacturer’s instructions (Roche Diagnostics, Mannheim, Germany). All cultures were daily analyzed under a light microscope, checking for irregular growth patterns and detached cells. Furthermore, the histologic analysis of the long-term cultures revealed no microscopically detectable signs of cytotoxic effects.

### Culture of primary bovine osteoblasts (pOBs)

The bovine periosteal specimens were obtained from the tibias of 1.5-year-old cattle, and the pOBs were isolated and cultured as previously described [10, 11]. The periosteal samples were washed for more than 60 minutes in Earl’s balanced salt solution, followed by a second wash for 20 minutes in Earl’s balanced salt solution containing antibiotics (500 IU/ml penicillin, 50 μg/ml streptomycin, 0.02 mg/ml gentamycin and 0.004 mg/ml amphotericin B) at 37°C. Next, the periosteal specimens were transferred to standard plastic dishes (15 cm) with 15 ml of BGJb medium Fitton-Jackson modification and were incubated at 37°C at 5% CO_2_ in humidified air. The culture medium was renewed every seven days. At approximately 4 weeks, the cells reached confluency of 80% and were subcultured using accutase (PAA Laboratories GmbH, Cölbe, Germany).

### Collection of tendon specimens and culture of primary bovine fibroblasts (pFBs)

Bovine tendon specimens were obtained from the lower leg tendons of 1.5-year-old cattle. The attached soft tissue was removed, and the tendons were washed in Earl’s balanced salt solution for 30 minutes and cut into segments (0.5 x 0.5 cm). The specimens were washed again in modified Earl’s solution containing antibiotics. To isolate the pFBs, the tendon specimens were fixed with Tissuco fibrin glue (Tissucol, Baxter, Munich, Germany) in Lumox cell culture dishes (35 mm) and were covered with BGJb culture medium. The incubation of the specimens was performed for 4 weeks at 37°C, in 100% humidity and 5% CO_2_. During the outgrowth of cells, the medium was changed every 7 days. At approximately four weeks, the cells reached confluency, and accutase was applied to subculture the cells.

### Bone morphogenic protein-7 (BMP-7)

In the experimental groups treated with recombinant BMP-7 (Stryker, Mahwah, NJ, USA), the standard medium contained 400 ng/ml BMP-7 and was renewed every 48 hours.

### *In vitro* model I

To study the effects of recombinant BMP-7 on the biological processes at the tendon-bone interface, an *in vitro* model combining a coculture of vital bovine tendon specimens and pOBs was established. The bovine tendon specimens were obtained as previously described and were fixed to Lumox cell culture dishes with Tissucol fibrin glue (9 specimens per group) [12]. The specimens were covered with BGJb medium (5% fetal calf serum [FCS], 400 ng/ml vitamin D_3_, 1.2 mg/ml NaHCO_3_, 50 µg of streptomycin, 0.02 mg/ml gentamycin and 500 IU/ml penicillin). Then, 1.25x10^7^ vital pOBs per culture dish were seeded and cultured under the conditions described above. The medium was changed every 48 hours. Four experimental groups and one control group were studied:

- Monoculture of bovine tendon specimens without BMP stimulation (bT – BMP);
- Monoculture of bovine tendon specimens treated with 400 ng/ml BMP-7 (bT + BMP);
- Coculture of bovine tendon specimens and pOBs without BMP stimulation (pOB + bT – BMP);
- Coculture of bovine tendon specimens and pOBs treated with 400 ng/ml BMP-7
- pOB + bT + BMP);
- Monoculture of pOBs without BMP stimulation (pOB – BMP) as control group.

The experiment was terminated after 10 weeks (70 days).

Medium samples were obtained every 7 days and were stored at -80°C in 2-ml cups until further analyzed, and 24 hours before the medium samples were obtained, the medium was changed to a FCS-free medium. Every 28 days, three tendon specimens were obtained from each group for light-/electron-microscopic and biochemical analyses. For the cell extracts, the adherent cells were washed with ice-cold phosphate-buffered saline (PBS) and were removed by scraping. The cell pellets were homogenized in lysis buffer (203.3 mg/l MgCl_2_, 2422.8 mg/l TRIS, 13.6 mg/l ZnCl_2_, and 10% Triton X-100), using an Ultra-Turrax homogenizer (IKA, Staufen, Germany) at full speed. The cell lysates were transferred to 1.5-ml cups and were stored at -80°C.

### Histological examination

After 4, 8 and 10 weeks of cultivation, the tendons were harvested and prepared for histological examination. The specimens were fixed in 4% paraformaldehyde and were embedded in a paraffin solution. For further analysis, 6-µm-thick slices were used. Next, staining with von Kossa and Periodic Acid-Schiff reaction (PAS) was performed to visualize the calcification within the tendon specimens. The slides were blinded and independently analyzed by two investigators. Quantitative histological analysis of PAS stained tissue sections were performed under a 10-fold magnification. Absolute thickness of ECM formation characterized by apposition of collagen rich fibers was assessed on 10 randomly assigned areas of the tendon surface of each group. Measurements are given in μm.

### Electron microscopy (SEM)

The tendon specimens were fixed for 24 hours at 4°C in SEM-fixation buffer and then were washed in SEM buffer for 30 minutes. For secondary fixation, 4 hours in osmium tetroxide and a series of ethanol washes were performed. The specimens were rinsed in acetone, transferred to a critical point chamber (BAL-TEC GmbH, Witten, Germany) and dried with acetone and CO_2_. The dried specimens were transferred to the sputter coater, and their surfaces were coated with gold for the final analysis, using a JSM-7500F electron microscope (JEOL, Eching, Germany).

### In vitro model II

To focus on the cellular components of the bone-tendon interface and to minimize the biological effects mediated by ECM metabolism, an exclusively cell-based experimental design was established. To study the effects of BMP-7 stimulation on pOBs and pFBs, 6 experimental groups were analyzed:

- Monoculture pOBs without BMP-7 (pOB – BMP)
- Monoculture pOBs with 400 ng/ml BMP-7 (pOB + BMP)
- Monoculture pFBs without BMP-7 (pFB – BMP)
- Monoculture pFBs with 400 ng/ml BMP-7 (pFB + BMP)
- Coculture pOBs and pFBs without BMP-7 (pOB + pFB – BMP)
- Coculture pOBs and pFBs with 400 ng/ml BMP-7 (pOB + pFB + BMP)

The monocultures and cocultures were grown in Lumox cell culture dishes covered with 2.5 ml of BGJb-medium, and pOBs and pFBs were plated at an 80:20 ratio and at a density of 0.32x10^6^ cells/ml. A single application of BMP-7 was performed after 12 days. Every 96 hours, the medium was carefully removed, and the cell lysates were obtained. The experiment was terminated after day 24.

### Alkaline phosphatase (ALP) activity ELISA

For the measurement of the ALP activity, the cell pellets were homogenized in 0.5 ml of lysis buffer at 6°C. Following centrifugation at 14,000 rpm for 10 minutes, the supernatant was removed and stored at -20°C. The ALP activity was measured by commercially available ELISA (Roche Diagnostics). Following incubation with para-nitrophenylphosphate for 30 minutes, the pNA concentration was measured at a wavelength of 405 nm, using a microtiter plate photometer (Milenia, DPC, Los Angeles, CA, USA). For calibration, two standard ALP (BioRad, Irvine, CA, USA) activities (21.3 U/l and 75 U/l) were used. Absolute ALP activity was reported, relative ALP activity per cell was not analyzed.

### Lactate dehydrogenase (LDH) ELISA

To measure LDH activity, ELISA, based on the Warburg reaction, was applied (Roche Diagnostics). Following the addition of the reagents, the samples and standards were measured at a wavelength of 340 nm using a microtiter plate photometer. The standards were set at 40.3 U/l and 80 U/l, respectively.

### Lactate ELISA

The lactate concentration was measured using commercially available lactate ELISA (Roche Diagnostics). Following incubation with lactate oxidase and chromogen, the samples and standards were measured in a microtiter plate photometer at a wavelength of 405 nm. The standards were set at 4.22 mM and 1.11 mM, respectively.

### Osteocalcin ELISA

Commercially available osteocalcin (OCN) ELISA was used (Quidel Corporation, San Diego, CA, USA). Briefly, the samples and standards were measured in a 96-well plate. Following the addition of a monoclonal mouse anti-OCN antibody, the samples were incubated with conjugated anti-mouse antibodies. After incubation with a substrate solution, the samples were measured at a wavelength of 405 nm using a microtiter plate photometer.

### Statistical analysis

Student’s paired and unpaired t-tests and one-way analysis of variance (ANOVA) with post hoc turkey test were applied. P-values less than 0.05 were considered significant. Outliers were detected using a conventional outlier test and were eliminated accordingly. The statistical analyses were performed using MATLAB software (MathWorks, Ismaning, Germany) and SPSS software for Mac, version 22 (SPSS, Chicago, IL, USA).

## Results

### In vitro model I

**Figure 1:**
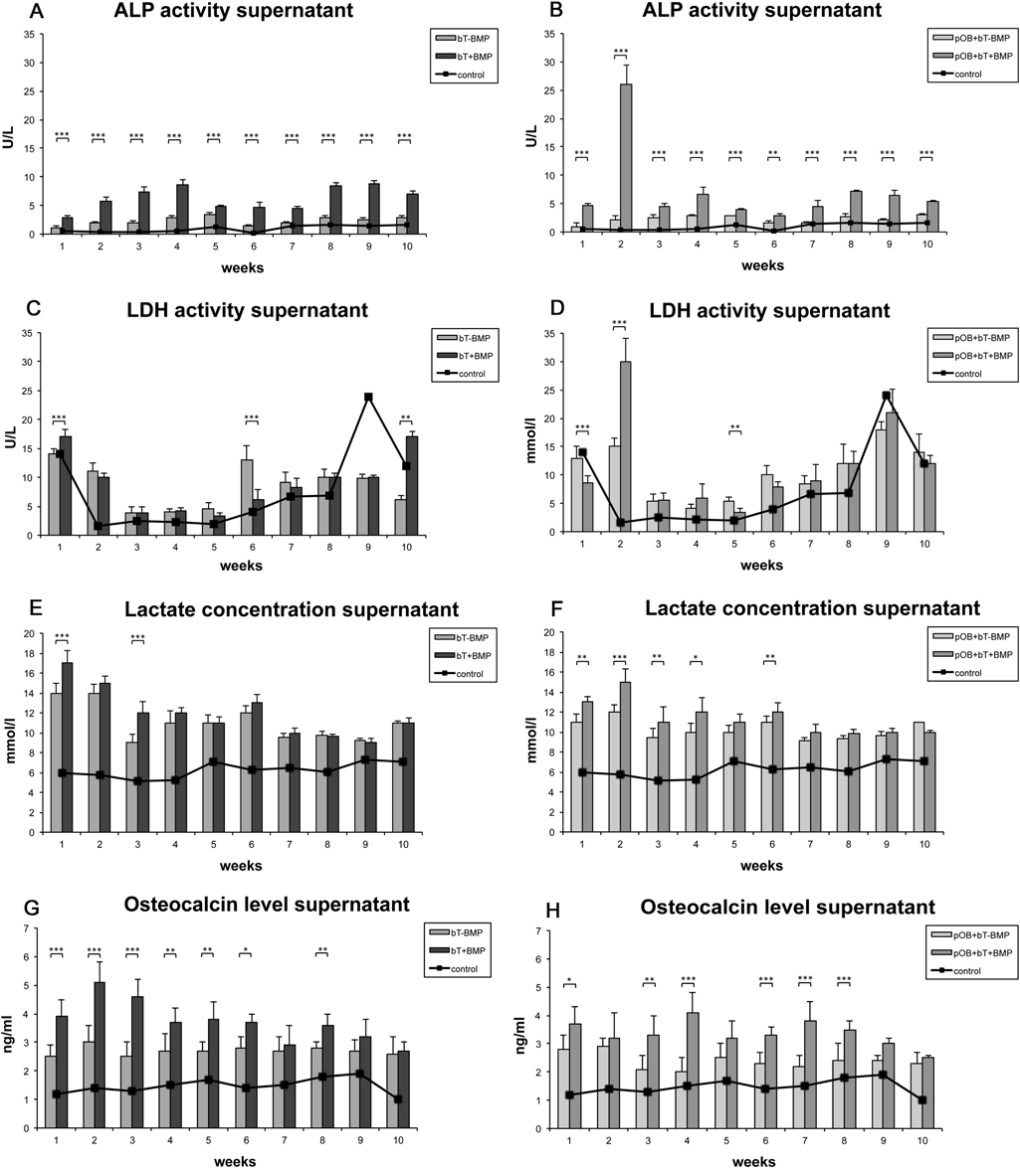
Diagram showing the effects of recombinant BMP-7 on alkaline phosphatase (ALP), lactate dehydrogenase (LDH), lactate and osteocalcin (OCN) (supernatant), combining the following four distinct groups: monoculture of bovine tendon specimens without BMP stimulation (bT-BMP); monoculture of bovine tendon specimens treated with 400 ng/ml BMP-7 (bT+BMP); coculture of bovine tendon specimens and pOBs without BMP stimulation (pOB+bT-BMP); and coculture of bovine tendon specimens and pOBs treated with 400 ng/ml BMP-7 (pOB+bT+BMP). Monoculture of pOBs without BMP stimulation served as a control (horizontal line).(A) Application of BMP-7 increased the levels of ALP activity, compared to those in the non-stimulated group. (B) A maximum peak of ALP activity was found in the BMP-7-stimulated cocultures of bovine tendons and pOBs after 14 days. (C) The group-specific LDH activity is shown. (D) Parallel to the findings regarding ALP activity, the highest levels of LDH activity were found in the coculture of tendons and pOBs at two weeks. (E) A moderate increase in lactate was observed during the early phase following BMP-7 stimulation of the monocultures, whereas no significant effects of BMP-7 were found during the later stages. (F) The BMP-7-stimulated coculture showed a significant increase during the early phase. In contrast to the results of ALP and LDH activity, a time-dependent decrease in the lactate concentration was found in all the groups. (G) BMP-7 application showed a significant increase in OCN expression in the tendon monoculture during an early phase of the experiment (week 2). (H) The stimulated cocultures peaked at a later stage (week 4). The data are presented as mean ± standard deviation. The asterisks (*) indicate the significant differences between the stimulated and non-stimulated groups (*p<0.05, **p<0.01, ***p<0.001).

**Supernatant ALP activity analysis**. The effects of BMP-7 on the ALP activity in the supernatants of the cocultured bovine tendon specimens and pOBs were characterized. As a control, the ALP activities of the non-stimulated pOB-monocultures were analyzed. The results revealed increased levels of ALP activity following stimulation with recombinant BMP-7 (bT+BMP and pOB+bT+BMP) at almost all the time points over the 10-week period as compared to the non-stimulated groups (bT-BMP and pOB+bT-BMP) (Fig. 1A and B). The most increased level of ALP activity was found in the BMP-7-stimulated cocultures of the bovine tendons and pOBs (pOB+bT+BMP), with a maximum activity of 26 U/l after 14 days. The lowest ALP levels were detected in the tendon monocultures (Fig. 1A); however, significant upregulation of enzyme activity was detected following BMP-7 stimulation. A further subgroup analysis comparing bT-BMP vs. pOB+bT-BMP and bT+BMP vs. pOB+bT+BMP is shown in S1 Fig. Here, no differences between the non-stimulated tendon monocultures and the cocultures were found. Following BMP-7 stimulation significantly increased levels of ALP activity were detected at early time points in the cocultures of the bovine tendons and pOBs (pOB+bT+BMP) when compared to tendon monocultures. However, a trend towards a time-dependent increase in ALP activity was noted in stimulated tendon monocultures.

**Supernatant LDH analysis**. The effects of recombinant BMP-7 on cell metabolism were analyzed by measuring the LDH activity (Fig. 1C and D). At most of the time points, no effects of BMP-7 on LDH activity were detectable. Parallel to the ALP activity, most of the increased levels of LDH activity were found in the cocultures after two weeks, and BMP-7 led to a significant increase in LDH activity (p<0.001). A trend toward a time-dependent increase in LDH activity was noted in all groups (Fig. 1C and D). A further subgroup analysis comparing bT-BMP vs. pOB+bT-BMP and bT+BMP vs. pOB+bT+BMP is shown in S1 Fig.

**Supernatant lactate concentration**. To further investigate the metabolic status of the analyzed cultures, the lactate concentrations in the supernatants were measured (Fig. 1E and F). There was an increase in lactate in the first two weeks following BMP-7 stimulation, whereas no effects of BMP-7 were found at the later time points. A time-dependent decrease in the lactate concentration was found in all of the groups. A further subgroup analysis comparing bT-BMP vs. pOB+bT-BMP and bT+BMP vs. pOB+bT+BMP is shown in S1 Fig.

**Supernatant OCN analysis**. The osteoblastic differentiation of the stimulated and non-stimulated cultures was determined by the OCN concentration (Fig. 1G and H). BMP-7 stimulation led to an increase in the OCN protein concentration in the tendon monocultures, as well as in the cocultures. Whereas the effects of BMP-7 in tendon monoculture peaked during an early phase of the experiment (week 2), the cocultures showed a maximum increase in the OCN concentration during a later stage (week 4). At the end of the experiment, no differences between the stimulated and non-stimulated cultures were found. A further subgroup analysis comparing bT-BMP vs. pOB+bT-BMP and bT+BMP vs. pOB+bT+BMP is shown in S1 Fig. Here, no differences between the non-stimulated tendon monocultures and the cocultures were found. At early time points following the stimulation with BMP-7, significantly increased OCN levels were detected in the tendon monocultures when compared to the cocultures with pOB.

**Figure 2:**
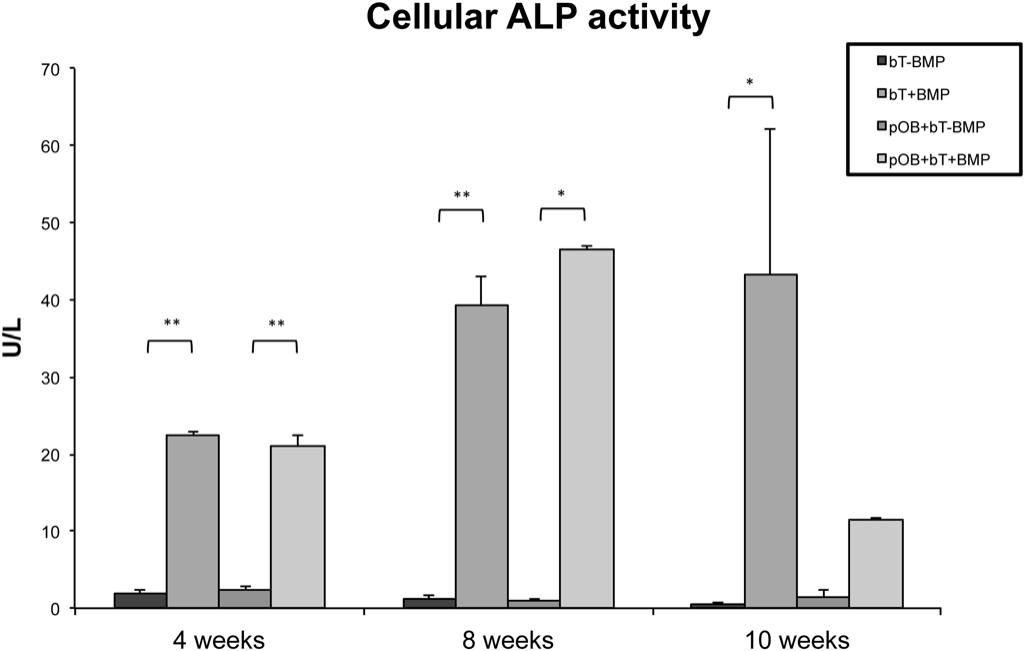
Diagram showing the cellular ALP activities of mono- and cocultures. Both BMP-7-stimulated groups (bT+BMP and pOB+bT+BMP) showed significantly increased ALP activities at all the time points as compared to the non-stimulated groups. Whereas the peak cellular ALP of the stimulated coculture (pOB+bT+BMP) was found at 8 weeks, there was a further increase in the ALP activity at 10 weeks in the tendon monoculture (bT+BMP). The data are presented as mean ± standard deviation. The asterisks (*) indicate the significant differences between the stimulated and non-stimulated groups; statistically significant differences were indicated at all of the time points (* p<0.05, ** p<0.001).

**Cellular ALP activity analysis**. Following the analysis of ALP activity in the supernatant, the intergroup differences in absolute cellular ALP activity were investigated. Both BMP-7-stimulated groups (bT+BMP and pOB+bT+BMP) showed increased ALP activity at all the time points investigated, compared to non-stimulated groups (p<0.05) (Fig. 2). Whereas the maximum cellular ALP activity in stimulated cocultures (pOB+bT+BMP) was found at 8 weeks, there was a further increase after 10 weeks in tendon monocultures (bT+BMP) (Fig. 2).

**Figure 3:**
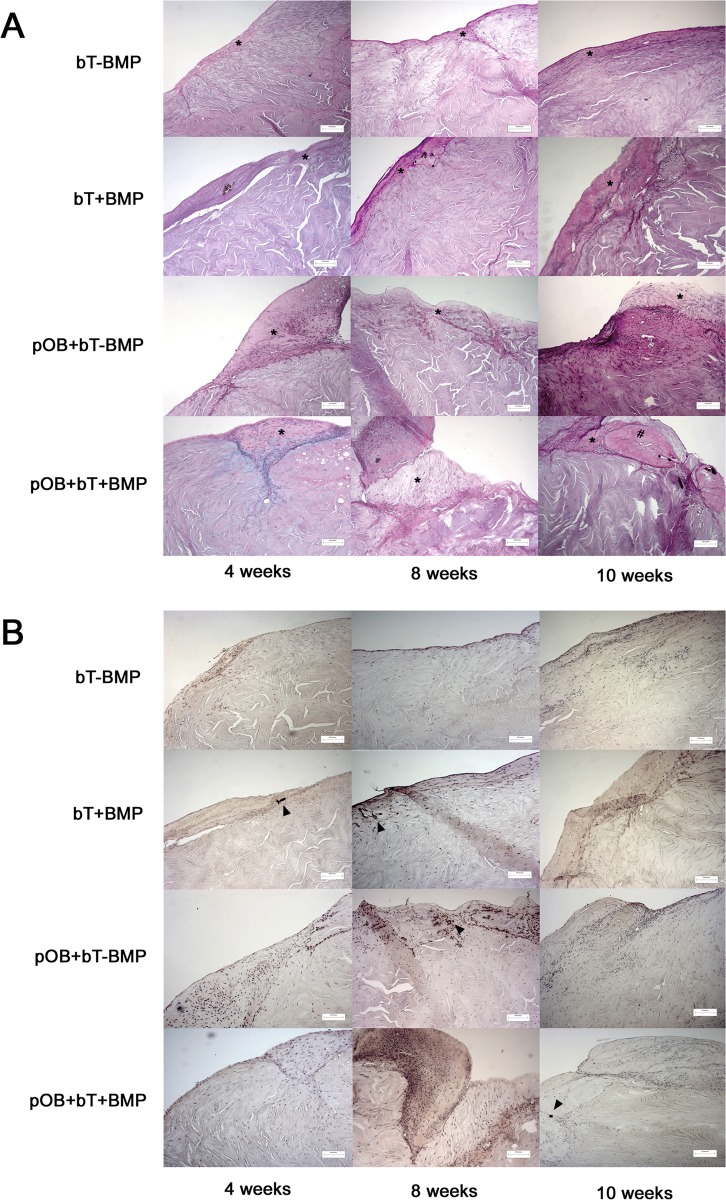
Histology images (PAS (A) and von Kossa (B) staining) under 10-fold magnification). The scale bars in the right column indicate 120 μm. The tendon specimens revealed an increased formation of extracellular matrix (*) after stimulation with BMP-7 at week 4 (bT+BMP and pOB+bT+BMP). Stimulation with BMP-7 led to intratendinous calcification (arrowhead). At 8 weeks, limited extracellular matrix formation was found in monoculture without BMP (bT-BMP), whereas coculture without BMP-7 (pOB+bt-BMP) showed increased rates of ECM formation and intratendinous calcification areas (arrowhead). Stimulation with BMP-7 further increased ECM formation and calcification compared to the specimens at week 4. At 12 weeks, coculture with BMP-7 application (pOB+bT+BMP) showed the highest amount of ECM (*), osteoid (#) and calcification (arrowhead), followed by monoculture with BMP-7 (bT+BMP) and coculture without BMP (pOB+bT-BMP). Calcification was detected in areas close to the extracellular matrix as shown in panel A in the pOB+bT-BMP group at 10 weeks. Monoculture without BMP (bt-BMP) showed limited ECM and intratendinous calcified tissue.

**Figure 4:**
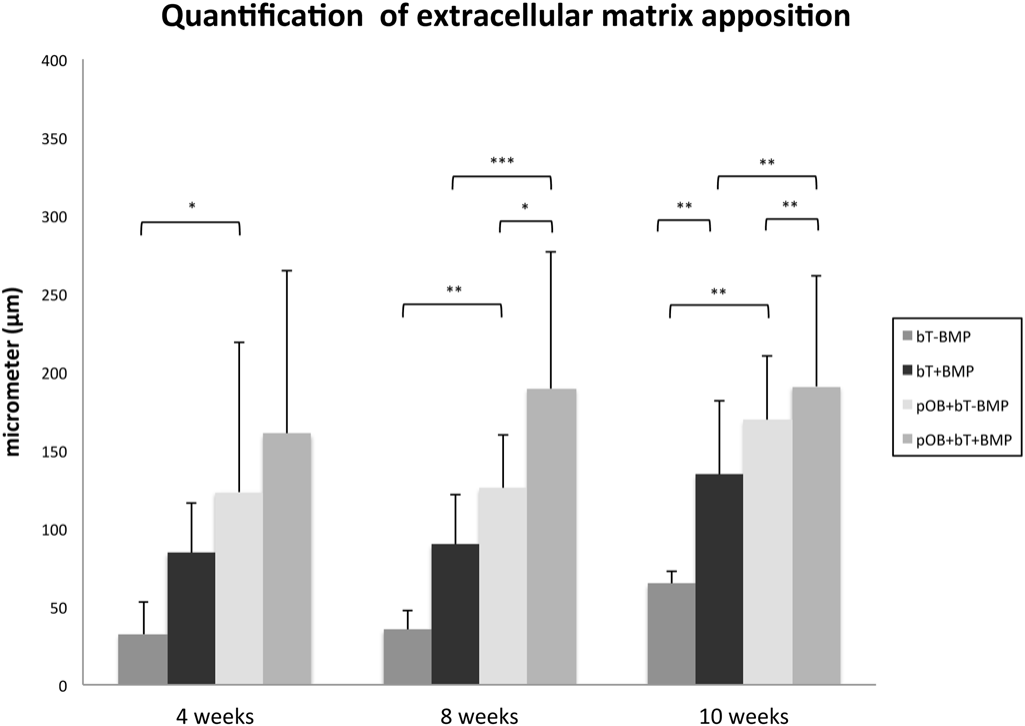
Quantitative histological analysis of extracellular matrix (ECM) formation. Thickness of group-specific extracellular matrix formation characterized by collagen rich fibers was measured in μm on histology specimens (PAS) under 10-fold magnification. Values are presented as mean ± standard deviation. The asterisks (*) indicate the significant differences between the stimulated and the non-stimulated groups (*p<0.05, **p<0.01, ***p<0.001).

**Histological findings**. The histological analyses of the tendon specimens revealed increased formation of ECM after stimulation with BMP-7 at 4 weeks (Figs. 3A and B and 4). BMP-7 exposure led to intratendinous calcification, which was not detectable in the non-stimulated specimens (Fig. 3A and B). At 8 weeks, limited ECM formation was found in monoculture without BMP (bT-BMP), whereas the coculture (pOB+BT-BMP) showed increased ECM formation and intratendinous calcification. Stimulation with BMP-7 further increased ECM formation and calcification at 4 weeks. This trend continued at 10 weeks, with the greatest amounts of ECM and calcification found in coculture with BMP-7 application (pOB+bT+BMP), followed by monoculture with BMP application (bT+BMP) and coculture without BMP-7 (pOB+bT-BMP), whereas minimal ECM and no calcification were found in monoculture without BMP (bT-BMP) (Figs. 3A and B and 4).

**Figure 5:**
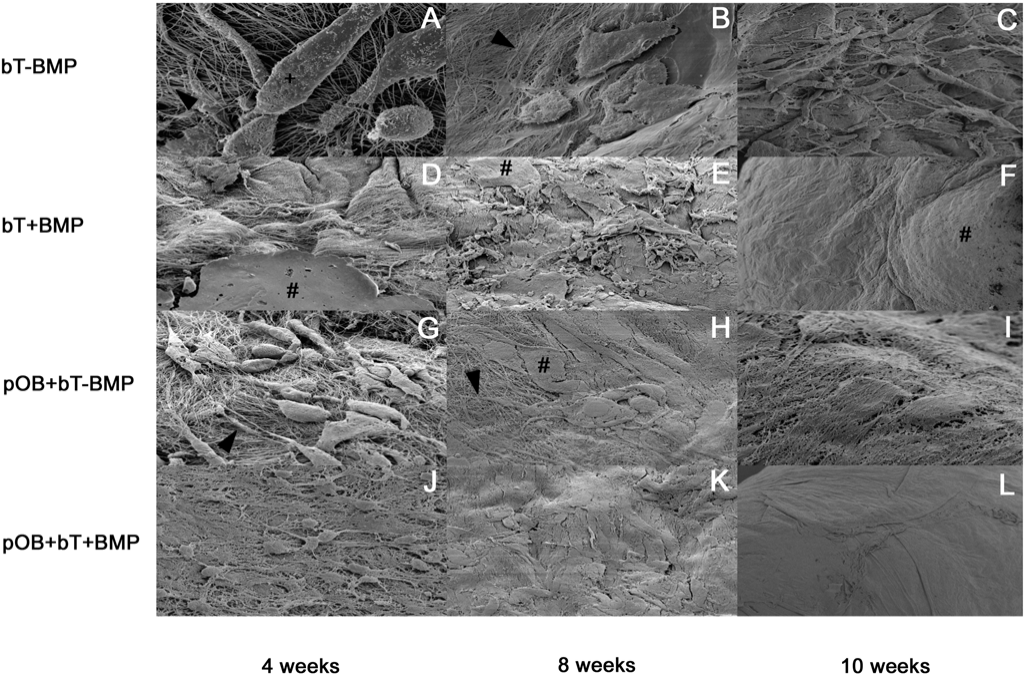
Scanning electron microscope (SEM) images of groups 1–4 (*in vitro* model I) at weeks 4, 8 and 10. (A-C) Monoculture without BMP (bT-BMP) showed the lowest concentrations of collagen fibrils (arrowhead) and ECM plaques (#) at the three time points. (D-F) BMP-7 stimulation led to increased denseness of collagen fibrils at the surface, to greater density of collagen fibrils and to increased formation of ECM plaques. (G-I) In the non-stimulated coculture, increased density of collagen fibrils and increased homogeneity at the surface after 10 weeks could be observed as compared to monoculture without BMP (bT-BMP). (J-L) The BMP-7-stimulated coculture already showed the greatest density of collagen fibrils after 4 weeks. At 10 weeks, the tendons were completely covered with homogeneous ECM. (+) indicates cell body.

**Scanning electron microscopy (SEM)**. At 4 weeks, SEM revealed increased density of collagen fibrils at the surface of the BMP-7-stimulated tendons, compared to that in the non-stimulated groups. Monoculture without BMP-7 (bT-BMP) showed the lowest concentration of collagen fibrils and ECM plaques at the three time points (Fig. 5 A–C). Stimulation with BMP-7 led to increased density of collagen fibrils and ECM plaque formation. Coculture with pOBs further stimulated the formation of ECM plaques and increased the density of collagen fibrils, leading to increased numbers of detectable cellular structures. At 10 weeks, the collagen network was visible in mono- and coculture without BMP-7 (bT-BMP; pOB+bT-BMP) (Fig. 5C and I), whereas the tendons of the stimulated groups were completely covered (Fig. 5F and L).

### *In vitro* model II

**Figure 6:**
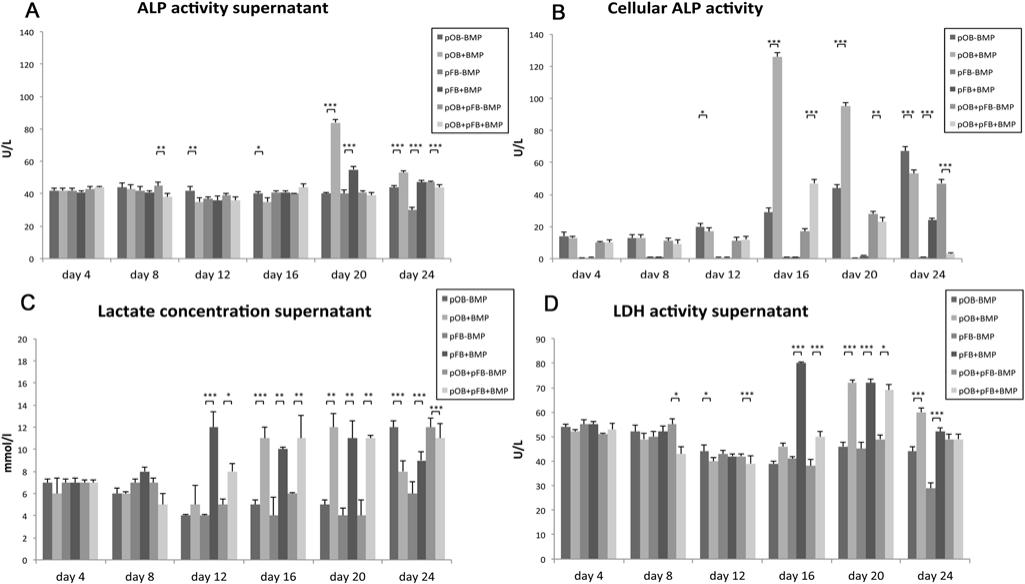
The effects of BMP-7 stimulation on mono- and cocultures of primary bovine osteoblasts and fibroblasts, combining the following 6 experimental groups: monoculture pOBs without BMP-7 (pOB-BMP); monoculture pOBs with 400 ng/ml BMP-7 (pOB+BMP); monoculture pFBs without BMP-7 (pFB-BMP); monoculture pFBs with 400 ng/ml BMP-7 (pFB+BMP); coculture pOBs and pFBs without BMP-7 (pOB+pFB-BMP); and coculture pOBs and pFBs with 400 ng/ml BMP-7 (pOB+pFB+BMP). (A) The BMP-7 application significantly increased the ALP activity in the pFB and pOB monocultures from day 20, whereas no significant effects were found in the coculture groups. (B) Stimulation with BMP-7 led to a significant increase in the cellular ALP activity in the pOB monocultures and cocultures. At the end of the experiment, a significant increase in cellular ALP activity was detectable following BMP-7 stimulation in the fibroblast monoculture. (C) The lactate concentration significantly increased in all groups following a delay of 12 to 16 days after BMP-7 stimulation. At the end, a decrease or an inversion of the effect of BMP-7 was found. (D) BMP-7 application resulted in significantly increased LDH activity in the pFB monoculture. At day 20, the stimulated groups showed significantly higher LDH activity. The data are presented as mean ± standard deviation. The asterisks (*) indicate the significant differences between the stimulated and the non-stimulated groups (*p<0.05, **p<0.01, ***p<0.001).

**Supernatant ALP activity analysis**. No relevant differences in the ALP activities between BMP-7 stimulated and non-stimulated cultures were detectable at the beginning of the experiment (Fig. 6A). From day 20, stimulation with BMP-7 led to a significant increase in ALP activity in the pFB and pOB monocultures (pOB+BMP and pFB+BMP). No significant effects of BMP-7 stimulation were found in the coculture group. Differences between both mono- and the coculture with and without BMP-7 exposure are shown in S2 Fig.

**Cellular ALP activity analysis**. Decreased cellular ALP activity was found in the pFB monocultures at all time points (Fig. 6B). From day 16, stimulation with BMP-7 led to a significant increase in cellular ALP activity in the pOB monocultures and cocultures. At day 24, a significant increase was detectable in cellular ALP activity following BMP-7 stimulation in the pFBs, whereas an inverse effect of BMP-7 on pOBs was noted. Differences between both mono- and the coculture with and without BMP-7 exposure are shown in S2 Fig.

**Supernatant LDH analysis**. The analysis of the LDH activity in the supernatants revealed no significant effects of BMP-7 stimulation until day 12 (Fig. 6D, S2 Fig.). Subsequently, significant increases in the LDH activity in the fibroblast monocultures and cocultures were found. From day 20, BMP-7 application resulted in increased LDH activity in pOBs as well. On days 12, 16 and 20, BMP-7 exposure led to a significant increase in the LDH activity in the coculture group. Differences between both mono- and the coculture with and without BMP-7 exposure are shown in S2 Fig.

**Supernatant lactate concentration**. The lactate concentration increased in all groups after 12 to 16 days following BMP-7 stimulation (Fig. 6 C). At day 24, a decrease or an inversion of the effect of BMP-7 was found. Differences between both mono- and the coculture with and without BMP-7 exposure are shown in S2 Fig.

## Discussion

Approximately 100,000 ACL reconstructions are annually performed in the USA, emphasizing the socioeconomic effects of this procedure [13]. An optimal clinical outcome depends on successful integration of the autologous tendon graft into the osseous tunnel [14]. During the healing process, biological interactions between calcified and non-calcified tissue subtypes are vitally important [15]. Rodeo et al. demonstrated correlations of degree of graft ingrowth and re-mineralization with the biomechanical strength of the tendon-bone interface [16].

Members of the BMP family have been shown to enhance the biological process of tendon ingrowth through the stimulation of bone formation at the bone-tendon interface [17]. BMP-7 has been shown to upregulate cellular proliferation in ovine ACL grafts, resulting in areas of dense trabecular network and enhancement of the invasion of fibrous tissue into the osseous structures [18, 19]. The majority of previous experimental studies have focused on the effects of BMP on the histological parameters of bone-tendon integration at defined time points, whereas the dynamics of the effects of BMP-7 have not been studied in depth. After establishing a long-term coculture model of pOBs and tendons, we were able to analyze the biological effects of BMP-7 stimulation, and we hypothesized that BMP-7 would enhance the biological processes associated with graft incorporation *in vitro*.

### ALP activity

BMP-7 stimulation led to increased ALP enzyme activity over the entire period of the experiment. The maximum effect was observed in the cocultures of bovine tendons and pOBs after two weeks of culturing. This early peak might be explained by the induction of osteoblast differentiation by Runx2-dependent unfolded protein response (UPR) transducers such as ATF6, a basic leucine zipper domain transcription factor within the BMP signaling cascade [20, 21]. The subsequent reduction in ALP activity could be explained by the following negative feedback processes [22, 23]: BMP inhibitors, such as noggin and chordin, could have been released by osteoblast-like cell fractions; these antagonists displace BMP from its receptor and block the processes that lead to transcription of BMP-target genes. This negative feedback mechanism represents an important process that prevents overwhelming ossification by BMP signaling [22, 24–26]. Compared to noggin, sclerostin is characterized by less specific affinity for particular members of the BMP family; it antagonizes approximately all the members of the BMP subgroups. Whereas classical extracellular antagonists interfere in the early phases of the mineralization process, sclerostin is not expressed by osteoblasts until the late stages of mineralization [26]. The use of BMP heterodimers represents a possibility to avoid this antagonism. Zhu et al. showed that BMP-2/7 heterodimers were more potent inducers of osteoblast differentiation than homodimers, associated with less expression of noggin [26].

We observed significant upregulation of ALP activity in the BMP-7-stimulated tendon monocultures, indicating transdifferentiation processes in the tendon specimens. This finding was supported by results from other investigators, demonstrating that primary mouse skin fibroblasts were able to differentiate into osteoblastic-like matrix mineralizing cells following stimulation with BMP-2 and -7 [27, 28].

### Lactate and LDH analysis

Lactate and LDH served as markers for cellular metabolism. During the early phases, a significant increase in LDH activity was found following application of BMP-7 in both culture systems. Additionally, an increase in LDH activity was found in the fibroblast monocultures. Our data were supported by previous reports on the stimulation of metabolic processes by BMP-7. Thus, BMP-7 functioned as a key regulator of mitochondrial concentration in adipocytes and had significant effects on the overall energy expenditure in fat tissue [29]. BMP-7 influenced the LDH activity in ectodermal cell cultures, such as cerebral cortical cultures. Here, modulatory effects on cell death decisions were suggested [30]. Importantly, besides their important role in anaerobic cell metabolism [31, 32], LDH and lactate are well established markers for processes involving cytotoxicity and cytolysis [33].

### Osteocalcin analysis

OCN is typically secreted solely by osteoblasts, and it is used as a marker for the late stages of osteoblast differentiation [34]. In our study, BMP-7 increased the OCN expression levels in all the subgroups. In contrast to other investigators, we found an increase in the OCN levels in BMP-stimulated tendon monocultures [35]. Wang et al. reported cell transdifferentiation of fibroblasts in an osteoblast/fibroblast coculture model [2]. Our results might indicate that BMP-7 could induce the expression of osteoblast-specific genes and the formation of calcified tissue of tenocytes/fibroblasts, even without the presence of osteoblasts. Here further experimental studies focusing on BMP-7 triggered transdifferentiation processes are required.

### Histology/scanning electron microscopy

After 4 weeks of cultivation, increased formation of ECM and calcified areas were observed in the BMP-7-stimulated groups, whereas the non-stimulated groups showed less ECM and no evidence of calcification. At 8 weeks, limited ECM formation was found in the tendon monoculture without BMP-7, whereas the BMP-stimulated coculture showed the highest rates of ECM formation and intratendinous calcification. This trend continued at 10 weeks. Interestingly, we were not able to show calcification within the extracellular matrix. Although calcification was described in *in vivo* studies at earlier time points no convincing data is available for *in vitro* studies. One possible explanation may be that calcification occurs later under *in vitro* conditions and was not included in the time points investigated in the current study. Scanning electron microscopy confirmed histological results that were in accordance with our findings regarding the regulation of OCN and ALP.

Increased ECM formation was detected at the tendon boundary region following stimulation with BMP-7. This effect became particularly obvious after 10 weeks, emphasizing the effects of BMP-7 on ECM synthesis and metabolism. Based on our results, BMP-7 appeared to be a relevant modulator of fibroblast/tendinocyte metabolism and gene expression, in addition to its well-characterized effects on osseous cells and tissues.

The primary limitations of this study were the lack of *in vivo* data and the focus on a single member of the BMP family. Comparative analyses, including other growth factors and the investigation of their interactions, are required. Confounding effects resulting from the long-term *in vitro* cell culture must be anticipated. While no quantitative tests for cell viability were performed, all long-term cultures were daily analyzed under a light microscope. No signs of irregular growth patterns or relevant numbers of detached cells were noted. Furthermore, the histologic analyses of the long-term cultures revealed no microscopically detectable signs of cytotoxic effects, (i.e. no relevant number of apoptotic cells displaying chromatin condensation) were identified.

Another potential drawback of the present study might be the inhomogeneous exposure to BMP-7 between experiment I (repetitive exposure) and experiment II (single exposure). First we established the long term-mono- and coculture of tendon specimens (*in vitro* model I) and applied BMP-7 repetitively to maintain a constant concentration possibly maximizing its biologic effects. Following the encouraging results obtained from *in vitro* model I, a protocol involving a single application of BMP-7 was chosen for the solely cell-based *in vitro* model II. Furthermore, the single exposure of BMP-7 led to an improved comparability to the currently established clinical treatment protocols using BMP-7 [36–38].

## Conclusions

Our long-term *in vitro* study supported the hypothesis that BMP-7 stimulation would induce beneficial effects on the bone-tendon integration process *in vivo*. BMP-7 was found to contribute to surrogates of osseous integration between tendon and bone by triggering the mineralization process. In addition to the well known influences of BMP-7 on bovine osteoblasts, we detected effects of BMP-7 on bovine tendons/fibroblasts, including the induction of osteocalcin expression. The clinical relevance of these observations in ACL surgery in humans has yet to be proved; however, the results of our *in vitro* cell culture model suggested the possibility of improved surgical outcomes. Further studies, including analyses of the native expression levels of BMP members at the bone-tendon interface *in vivo*, might improve our understanding of the role of BMPs in the process of bone-tendon healing.

## Supporting Information

S1 Fig Subgroup specific diagrams of experiment 1 (see Fig. 1). The effect of recombinant BMP-7 on alkaline phosphatase (ALP), lactate dehydrogenase (LDH), lactate and osteocalcin (OCN) (supernatant) is shown. Monoculture of bovine tendon specimens without BMP stimulation (bT-BMP) and coculture of bovine tendon specimens and pOBs without BMP stimulation (pOB+bT-BMP), as well as monoculture of bovine tendon specimens treated with 400 ng/ml BMP-7 (bT+BMP) and coculture of bovine tendon specimens and pOBs treated with 400 ng/ml BMP-7 (pOB+bT+BMP) were compared. Monoculture of pOBs without BMP stimulation served as a control (horizontal line). The data are presented as mean ± standard deviation. The asterisks (*) indicate the significant differences between the stimulated and non-stimulated groups (*p<0.05, **p<0.01, ***p<0.001).
(TIF)

S2 Fig Subgroup specific diagrams of experiment 2 (see Fig. 6). The effect of BMP-7 stimulation on mono- and cocultures of primary bovine osteoblasts and fibroblasts on alkaline phosphatase (ALP) (cellular and supernatant), lactate dehydrogenase (LDH) and lactate concentration is shown. On the left, monoculture pOBs without BMP-7 (pOB-BMP); monoculture pFBs without BMP-7 (pFB-BMP); and coculture pOBs and pFBs without BMP-7 (pOB+pFB-BMP) are compared. On the right, monoculture pOBs with 400 ng/ml BMP-7 (pOB+BMP); monoculture pFBs with 400 ng/ml BMP-7 (pFB+BMP); and coculture pOBs and pFBs with 400 ng/ml BMP-7 (pOB+pFB+BMP) were compared. The data are presented as mean ± standard deviation. The asterisks (*) indicate the significant differences between the stimulated and non-stimulated groups (*p<0.05, **p<0.01, ***p<0.001).
(TIF)
