# Supplementary figures and images for: Bone Morphogenetic Protein 7 (BMP-7) Influences Tendon-Bone Integration In Vitro
Source: PLoS One. 2015 Feb 2;10(2):e0116833. doi: 10.1371/journal.pone.0116833 (PMC4314204; doi:10.1371/journal.pone.0116833)

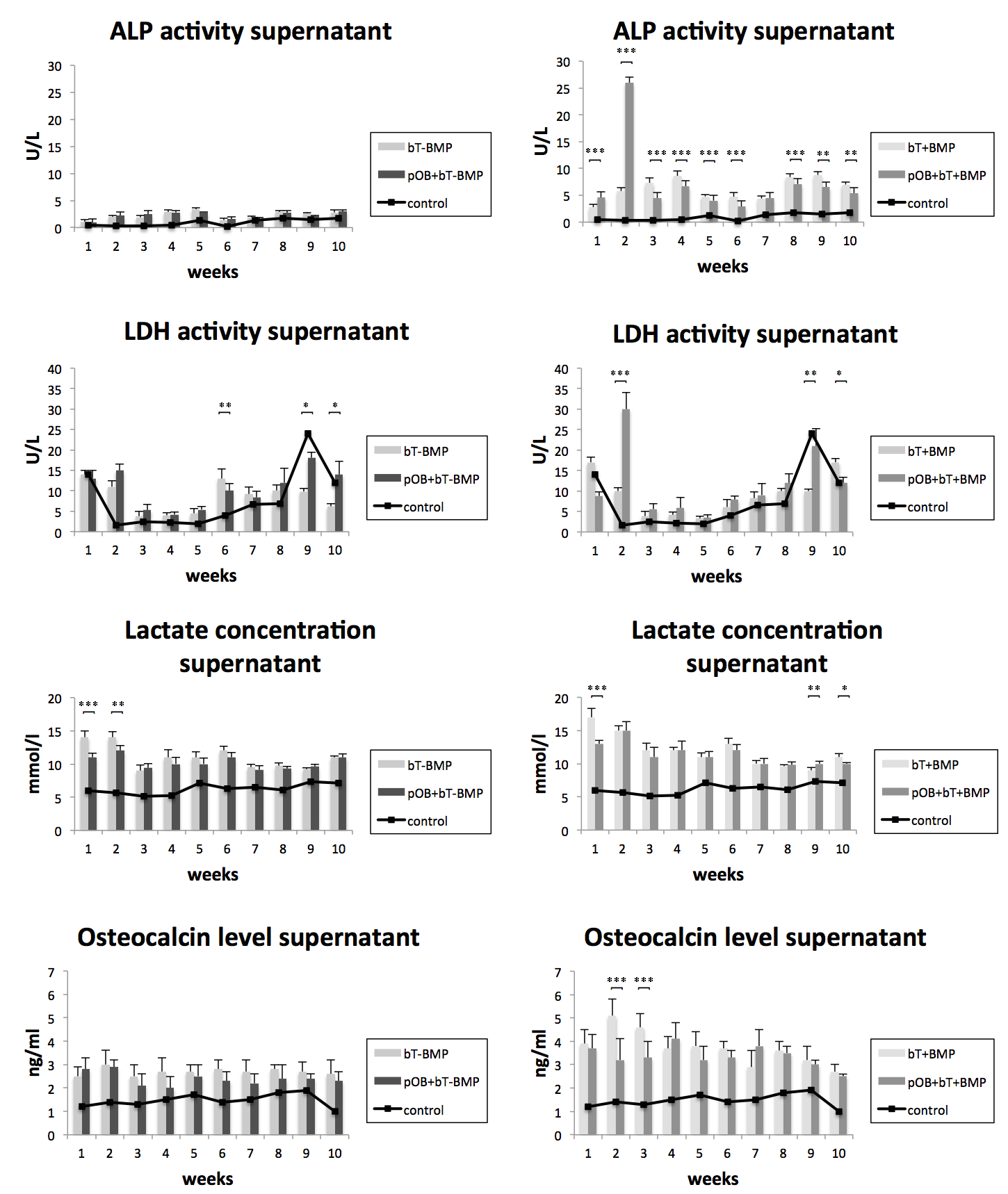

Supplement: S1 Fig — The effect of recombinant BMP-7 on alkaline phosphatase (ALP), lactate dehydrogenase (LDH), lactate and osteocalcin (OCN) (supernatant) is shown. Monoculture of bovine tendon specimens without BMP stimulation (bT-BMP) and coculture of bovine tendon specimens and pOBs without BMP stimulation (pOB+bT-BMP), as well as monoculture of bovine tendon specimens treated with 400 ng/ml BMP-7 (bT+BMP) and coculture of bovine tendon specimens and pOBs treated with 400 ng/ml BMP-7 (pOB+bT+BMP) were compared. Monoculture of pOBs without BMP stimulation served as a control (horizontal line). The data are presented as mean ± standard deviation. The asterisks (*) indicate the significant differences between the stimulated and non-stimulated groups (*p<0.05, **p<0.01, ***p<0.001). (TIF) [file pone.0116833.s001.tif]

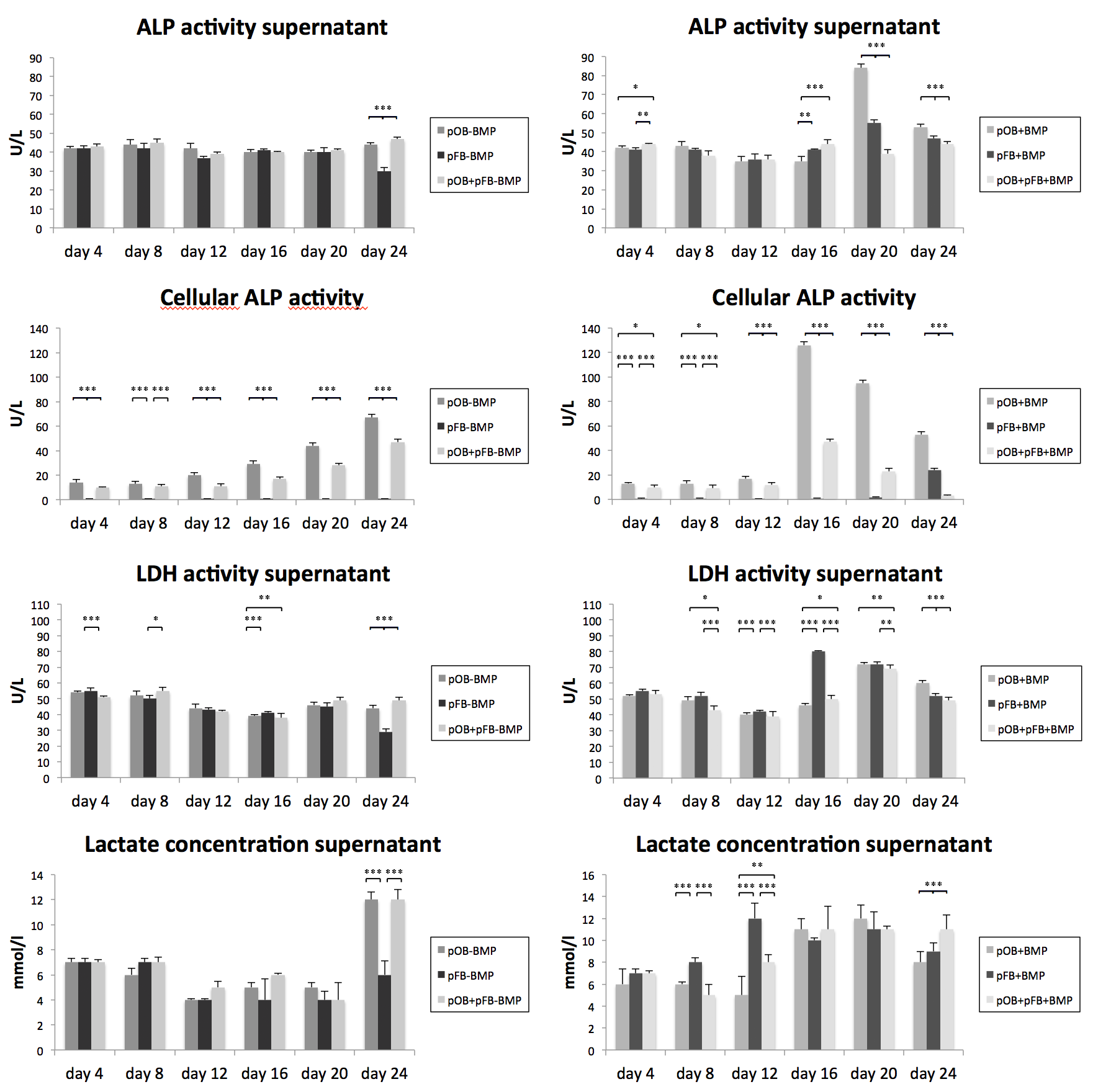

Supplement: S2 Fig — The effect of BMP-7 stimulation on mono- and cocultures of primary bovine osteoblasts and fibroblasts on alkaline phosphatase (ALP) (cellular and supernatant), lactate dehydrogenase (LDH) and lactate concentration is shown. On the left, monoculture pOBs without BMP-7 (pOB-BMP); monoculture pFBs without BMP-7 (pFB-BMP); and coculture pOBs and pFBs without BMP-7 (pOB+pFB-BMP) are compared. On the right, monoculture pOBs with 400 ng/ml BMP-7 (pOB+BMP); monoculture pFBs with 400 ng/ml BMP-7 (pFB+BMP); and coculture pOBs and pFBs with 400 ng/ml BMP-7 (pOB+pFB+BMP) were compared. The data are presented as mean ± standard deviation. The asterisks (*) indicate the significant differences between the stimulated and non-stimulated groups (*p<0.05, **p<0.01, ***p<0.001). (TIF) [file pone.0116833.s002.tif]
